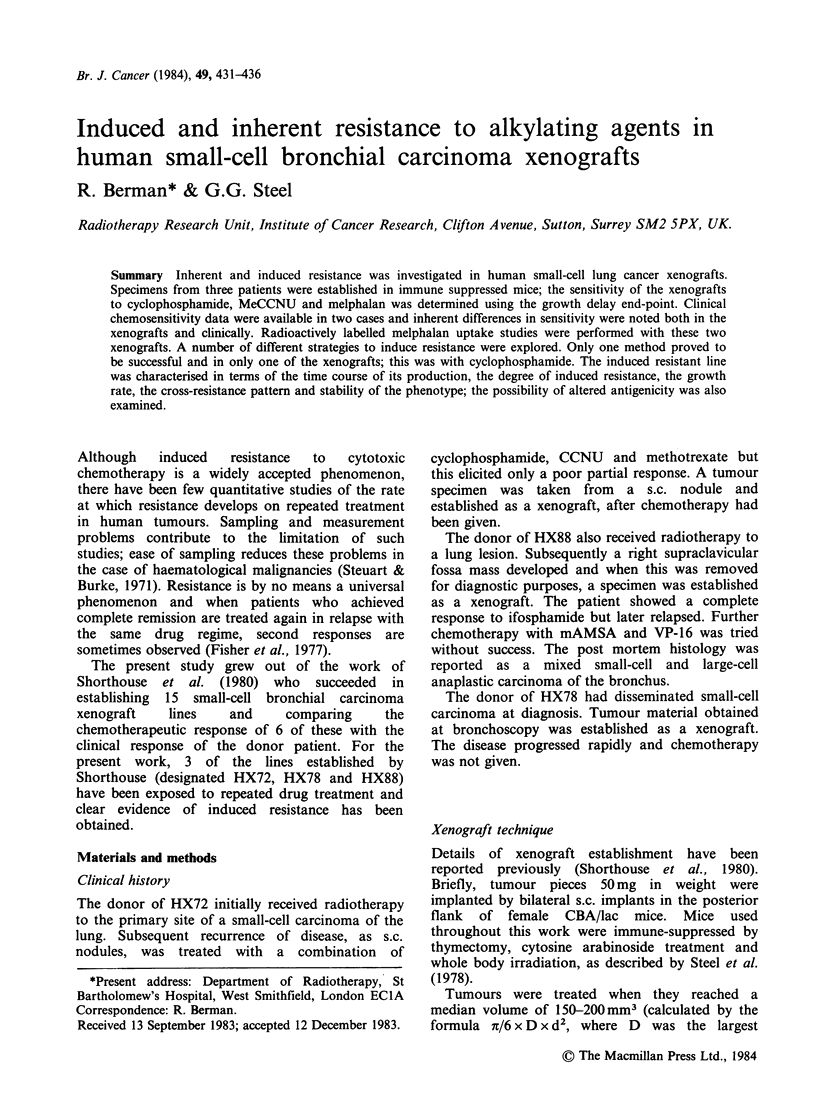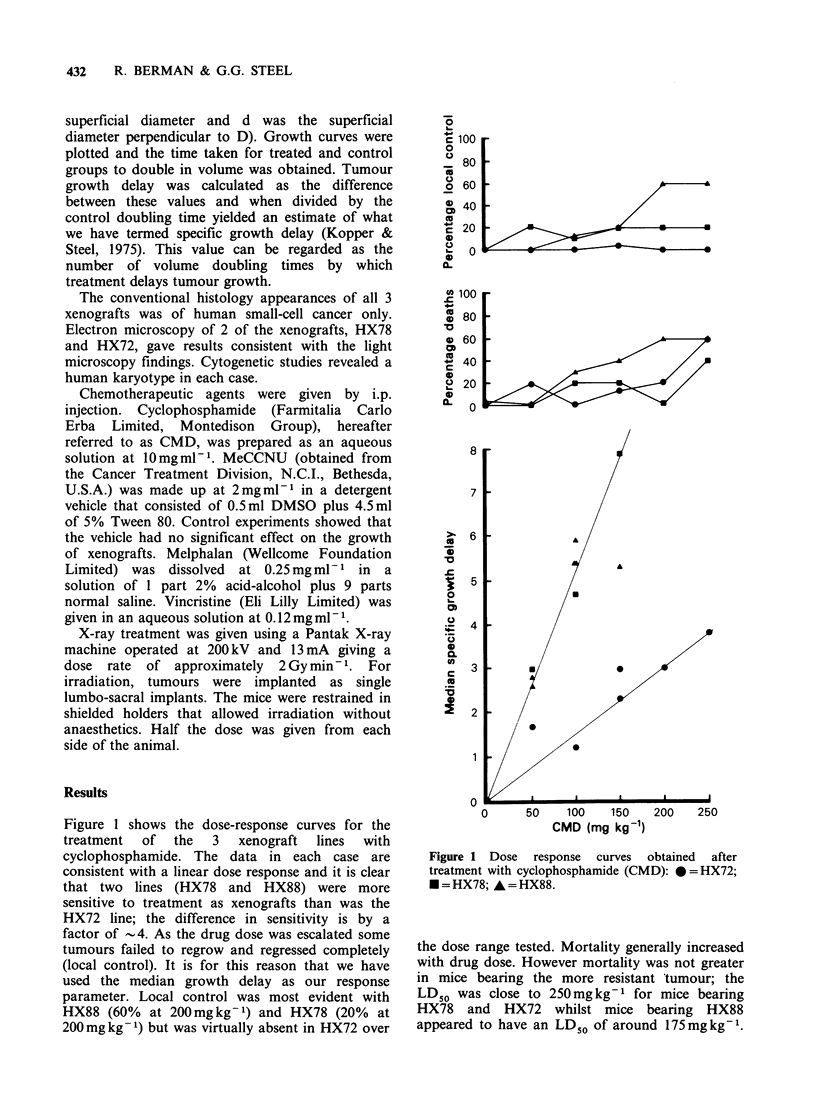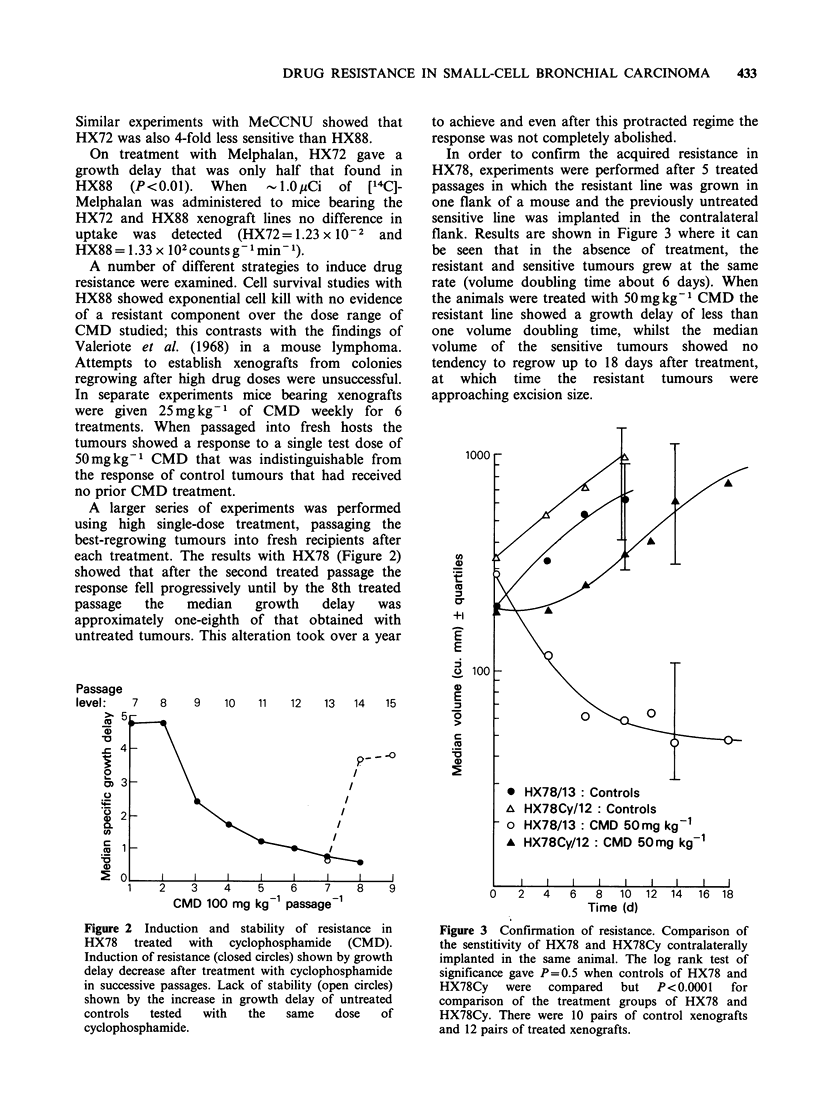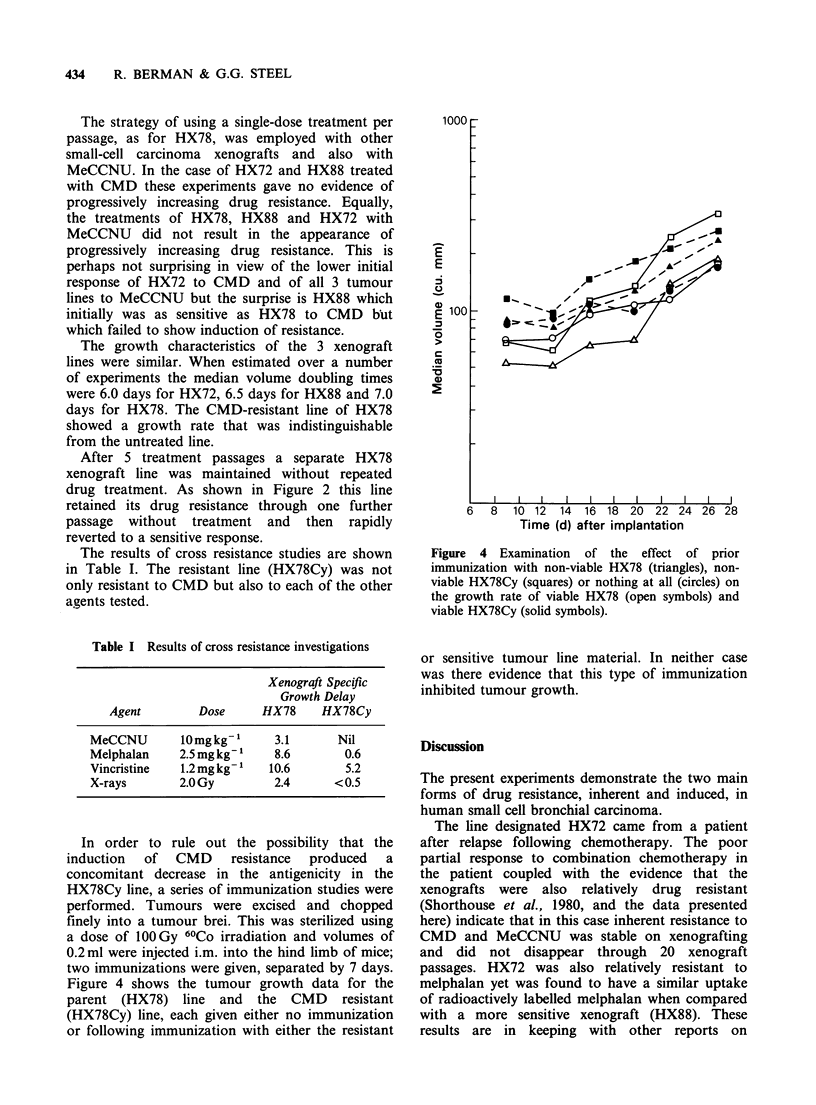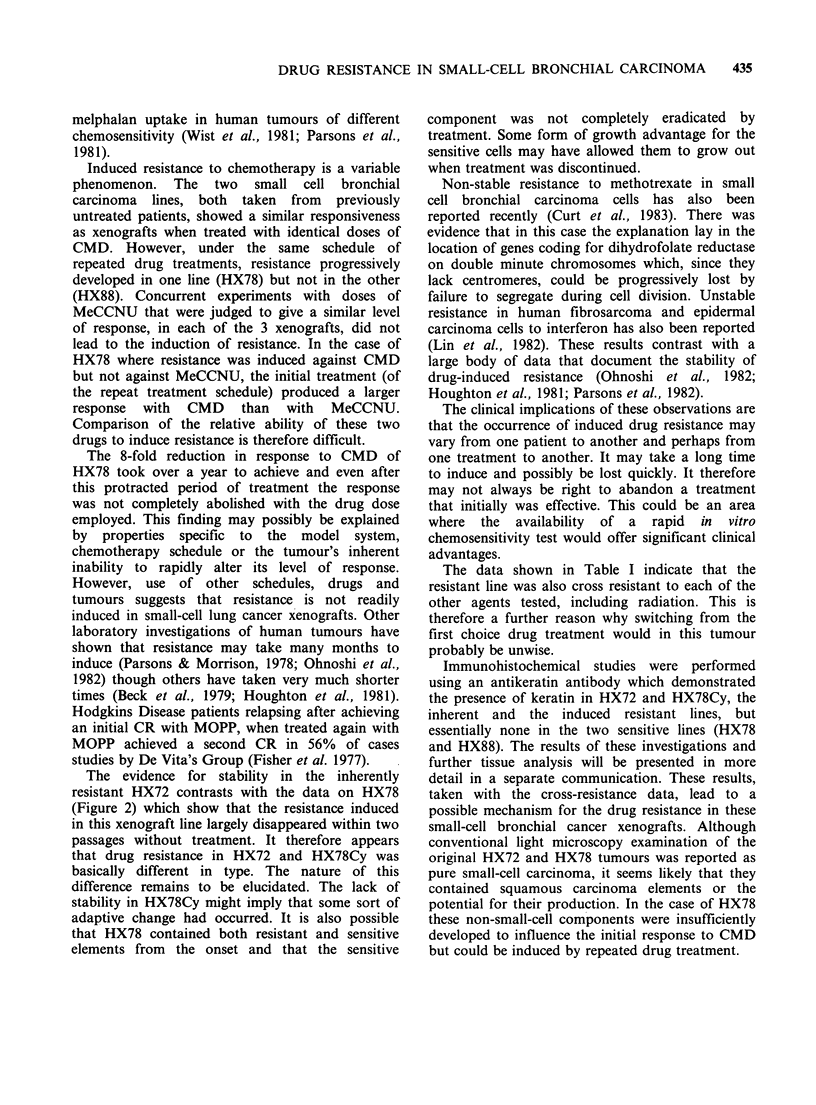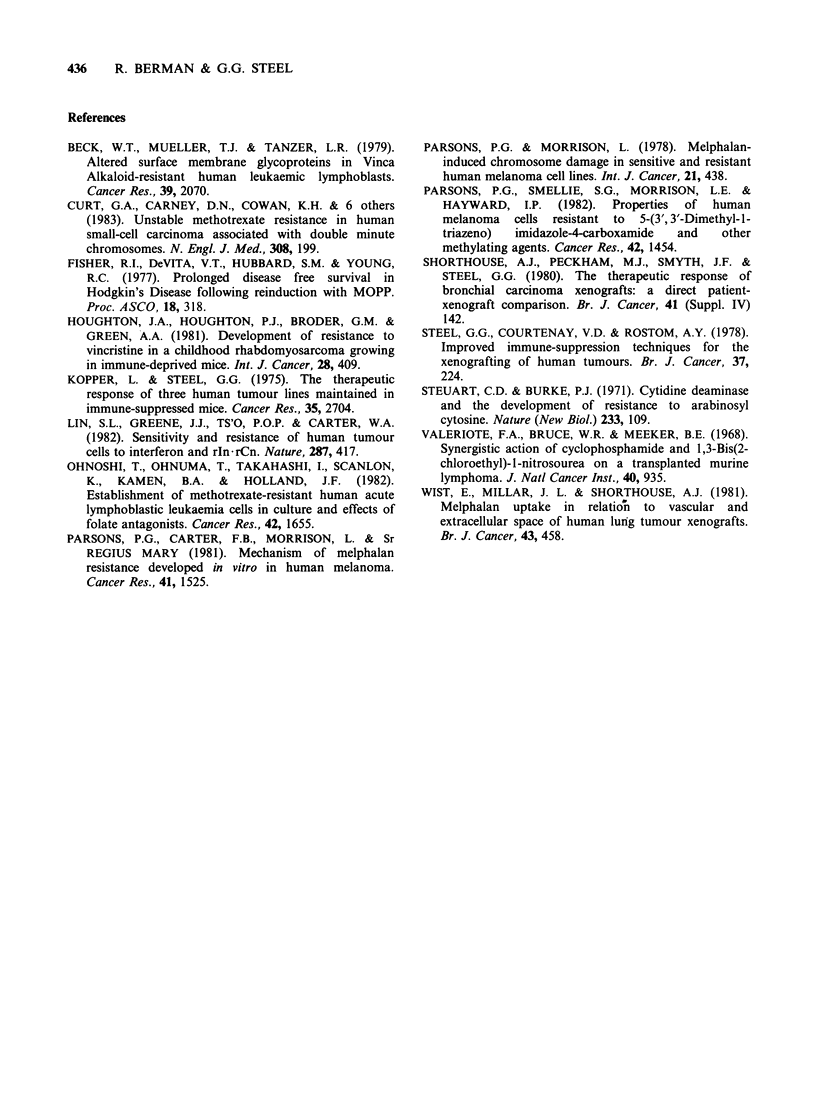# Induced and inherent resistance to alkylating agents in human small-cell bronchial carcinoma xenografts.

**DOI:** 10.1038/bjc.1984.69

**Published:** 1984-04

**Authors:** R. Berman, G. G. Steel

## Abstract

Inherent and induced resistance was investigated in human small-cell lung cancer xenografts. Specimens from three patients were established in immune suppressed mice; the sensitivity of the xenografts to cyclophosphamide, MeCCNU and melphalan was determined using the growth delay end-point. Clinical chemosensitivity data were available in two cases and inherent differences in sensitivity were noted both in the xenografts and clinically. Radioactively labelled melphalan uptake studies were performed with these two xenografts. A number of different strategies to induce resistance were explored. Only one method proved to be successful and in only one of the xenografts; this was with cyclophosphamide. The induced resistant line was characterised in terms of the time course of its production, the degree of induced resistance, the growth rate, the cross-resistance pattern and stability of the phenotype; the possibility of altered antigenicity was also examined.


*Br. J. Cancer* (1984), **49**, 431–436

### Induced and inherent resistance to alkylating agents in human small-cell bronchial carcinoma xenografts

R. Berman* & G.G. Steel

*Radiotherapy Research Unit, Institute of Cancer Research, Clifton Avenue, Sutton, Surrey SM2 5PX, UK.*

**Summary** Inherent and induced resistance was investigated in human small-cell lung cancer xenografts. Specimens from three patients were established in immune suppressed mice; the sensitivity of the xenografts to cyclophosphamide, MeCCNU and melphalan was determined using the growth delay end-point. Clinical chemosensitivity data were available in two cases and inherent differences in sensitivity were noted both in the xenografts and clinically. Radioactively labelled melphalan uptake studies were performed with these two xenografts. A number of different strategies to induce resistance were explored. Only one method proved to be successful and in only one of the xenografts; this was with cyclophosphamide. The induced resistant line was characterised in terms of the time course of its production, the degree of induced resistance, the growth rate, the cross-resistance pattern and stability of the phenotype; the possibility of altered antigenicity was also examined.

Although induced resistance to cytotoxic chemotherapy is a widely accepted phenomenon, there have been few quantitative studies of the rate at which resistance develops on repeated treatment in human tumours. Sampling and measurement problems contribute to the limitation of such studies; ease of sampling reduces these problems in the case of haematological malignancies (Steuart & Burke, 1971). Resistance is by no means a universal phenomenon and when patients who achieved complete remission are treated again in relapse with the same drug regime, second responses are sometimes observed (Fisher *et al.*, 1977).

The present study grew out of the work of Shorthouse *et al.* (1980) who succeeded in establishing 15 small-cell bronchial carcinoma xenograft lines and comparing the chemotherapeutic response of 6 of these with the clinical response of the donor patient. For the present work, 3 of the lines established by Shorthouse (designated HX72, HX78 and HX88) have been exposed to repeated drug treatment and clear evidence of induced resistance has been obtained.

### Materials and methods

### Clinical history

The donor of HX72 initially received radiotherapy to the primary site of a small-cell carcinoma of the lung. Subsequent recurrence of disease, as s.c. nodules, was treated with a combination of cyclophosphamide, CCNU and methotrexate but this elicited only a poor partial response. A tumour specimen was taken from a s.c. nodule and established as a xenograft, after chemotherapy had been given.

The donor of HX88 also received radiotherapy to a lung lesion. Subsequently a right supraclavicular fossa mass developed and when this was removed for diagnostic purposes, a specimen was established as a xenograft. The patient showed a complete response to ifosphamide but later relapsed. Further chemotherapy with mAMSA and VP-16 was tried without success. The post mortem histology was reported as a mixed small-cell and large-cell anaplastic carcinoma of the bronchus.

The donor of HX78 had disseminated small-cell carcinoma at diagnosis. Tumour material obtained at bronchoscopy was established as a xenograft. The disease progressed rapidly and chemotherapy was not given.

### Xenograft technique

Details of xenograft establishment have been reported previously (Shorthouse *et al.*, 1980). Briefly, tumour pieces 50 mg in weight were implanted by bilateral s.c. implants in the posterior flank of female CBA/lac mice. Mice used throughout this work were immune-suppressed by thymectomy, cytosine arabinoside treatment and whole body irradiation, as described by Steel *et al.* (1978).

Tumours were treated when they reached a median volume of 150–200 mm$^3$ (calculated by the formula $\pi/6 \times D \times d^2$, where D was the largest

*Present address: Department of Radiotherapy, St Bartholomew's Hospital, West Smithfield, London EC1A
Correspondence: R. Berman.

Received 13 September 1983; accepted 12 December 1983.

© The Macmillan Press Ltd., 1984

superficial diameter and d was the superficial diameter perpendicular to D). Growth curves were plotted and the time taken for treated and control groups to double in volume was obtained. Tumour growth delay was calculated as the difference between these values and when divided by the control doubling time yielded an estimate of what we have termed specific growth delay (Kopper & Steel, 1975). This value can be regarded as the number of volume doubling times by which treatment delays tumour growth.

The conventional histology appearances of all 3 xenografts was of human small-cell cancer only. Electron microscopy of 2 of the xenografts, HX78 and HX72, gave results consistent with the light microscopy findings. Cytogenetic studies revealed a human karyotype in each case.

Chemotherapeutic agents were given by i.p. injection. Cyclophosphamide (Farmitalia Carlo Erba Limited, Montedison Group), hereafter referred to as CMD, was prepared as an aqueous solution at $10 mg ml^{-1}$. MeCCNU (obtained from the Cancer Treatment Division, N.C.I., Bethesda, U.S.A.) was made up at $2 mg ml^{-1}$ in a detergent vehicle that consisted of 0.5 ml DMSO plus 4.5 ml of 5% Tween 80. Control experiments showed that the vehicle had no significant effect on the growth of xenografts. Melphalan (Wellcome Foundation Limited) was dissolved at $0.25 mg ml^{-1}$ in a solution of 1 part 2% acid-alcohol plus 9 parts normal saline. Vincristine (Eli Lilly Limited) was given in an aqueous solution at $0.12 mg ml^{-1}$.

X-ray treatment was given using a Pantak X-ray machine operated at 200 kV and 13 mA giving a dose rate of approximately $2 Gy min^{-1}$. For irradiation, tumours were implanted as single lumbo-sacral implants. The mice were restrained in shielded holders that allowed irradiation without anaesthetics. Half the dose was given from each side of the animal.

### Results

Figure 1 shows the dose-response curves for the treatment of the 3 xenograft lines with cyclophosphamide. The data in each case are consistent with a linear dose response and it is clear that two lines (HX78 and HX88) were more sensitive to treatment as xenografts than was the HX72 line; the difference in sensitivity is by a factor of ~4. As the drug dose was escalated some tumours failed to regrow and regressed completely (local control). It is for this reason that we have used the median growth delay as our response parameter. Local control was most evident with HX88 (60% at $200 mg kg^{-1}$) and HX78 (20% at $200 mg kg^{-1}$) but was virtually absent in HX72 over the dose range tested. Mortality generally increased with drug dose. However mortality was not greater in mice bearing the more resistant tumour; the $LD_{50}$ was close to $250 mg kg^{-1}$ for mice bearing HX78 and HX72 whilst mice bearing HX88 appeared to have an $LD_{50}$ of around $175 mg kg^{-1}$.

**Figure 1** Dose response curves obtained after treatment with cyclophosphamide (CMD): ● = HX72; ■ = HX78; ▲ = HX88.

Similar experiments with MeCCNU showed that HX72 was also 4-fold less sensitive than HX88.

On treatment with Melphalan, HX72 gave a growth delay that was only half that found in HX88 ($P<0.01$). When $\sim 1.0\,\mu$Ci of [$^{14}$C]-Melphalan was administered to mice bearing the HX72 and HX88 xenograft lines no difference in uptake was detected ($HX72 = 1.23 \times 10^{-2}$ and $HX88 = 1.33 \times 10^{2}$ counts $g^{-1} min^{-1}$).

A number of different strategies to induce drug resistance were examined. Cell survival studies with HX88 showed exponential cell kill with no evidence of a resistant component over the dose range of CMD studied; this contrasts with the findings of Valeriote *et al.* (1968) in a mouse lymphoma. Attempts to establish xenografts from colonies regrowing after high drug doses were unsuccessful. In separate experiments mice bearing xenografts were given 25 mg $kg^{-1}$ of CMD weekly for 6 treatments. When passaged into fresh hosts the tumours showed a response to a single test dose of 50 mg $kg^{-1}$ CMD that was indistinguishable from the response of control tumours that had received no prior CMD treatment.

A larger series of experiments was performed using high single-dose treatment, passaging the best-regrowing tumours into fresh recipients after each treatment. The results with HX78 (Figure 2) showed that after the second treated passage the response fell progressively until by the 8th treated passage the median growth delay was approximately one-eighth of that obtained with untreated tumours. This alteration took over a year to achieve and even after this protracted regime the response was not completely abolished.

In order to confirm the acquired resistance in HX78, experiments were performed after 5 treated passages in which the resistant line was grown in one flank of a mouse and the previously untreated sensitive line was implanted in the contralateral flank. Results are shown in Figure 3 where it can be seen that in the absence of treatment, the resistant and sensitive tumours grew at the same rate (volume doubling time about 6 days). When the animals were treated with 50 mg $kg^{-1}$ CMD the resistant line showed a growth delay of less than one volume doubling time, whilst the median volume of the sensitive tumours showed no tendency to regrow up to 18 days after treatment, at which time the resistant tumours were approaching excision size.

**Figure 2** Induction and stability of resistance in HX78 treated with cyclophosphamide (CMD). Induction of resistance (closed circles) shown by growth delay decrease after treatment with cyclophosphamide in successive passages. Lack of stability (open circles) shown by the increase in growth delay of untreated controls tested with the same dose of cyclophosphamide.

**Figure 3** Confirmation of resistance. Comparison of the senstitivity of HX78 and HX78Cy contralaterally implanted in the same animal. The log rank test of significance gave $P = 0.5$ when controls of HX78 and HX78Cy were compared but $P < 0.0001$ for comparison of the treatment groups of HX78 and HX78Cy. There were 10 pairs of control xenografts and 12 pairs of treated xenografts.

The strategy of using a single-dose treatment per passage, as for HX78, was employed with other small-cell carcinoma xenografts and also with MeCCNU. In the case of HX72 and HX88 treated with CMD these experiments gave no evidence of progressively increasing drug resistance. Equally, the treatments of HX78, HX88 and HX72 with MeCCNU did not result in the appearance of progressively increasing drug resistance. This is perhaps not surprising in view of the lower initial response of HX72 to CMD and of all 3 tumour lines to MeCCNU but the surprise is HX88 which initially was as sensitive as HX78 to CMD but which failed to show induction of resistance.

The growth characteristics of the 3 xenograft lines were similar. When estimated over a number of experiments the median volume doubling times were 6.0 days for HX72, 6.5 days for HX88 and 7.0 days for HX78. The CMD-resistant line of HX78 showed a growth rate that was indistinguishable from the untreated line.

After 5 treatment passages a separate HX78 xenograft line was maintained without repeated drug treatment. As shown in Figure 2 this line retained its drug resistance through one further passage without treatment and then rapidly reverted to a sensitive response.

The results of cross resistance studies are shown in Table I. The resistant line (HX78Cy) was not only resistant to CMD but also to each of the other agents tested.

**Figure 4** Examination of the effect of prior immunization with non-viable HX78 (triangles), non-viable HX78Cy (squares) or nothing at all (circles) on the growth rate of viable HX78 (open symbols) and viable HX78Cy (solid symbols).

**Table I** Results of cross resistance investigations

| Agent | Dose | *Xenograft Specific Growth Delay* *HX78* | *HX78Cy* |
|---|---|---|---|
| MeCCNU | $10\,mg\,kg^{-1}$ | 3.1 | Nil |
| Melphalan | $2.5\,mg\,kg^{-1}$ | 8.6 | 0.6 |
| Vincristine | $1.2\,mg\,kg^{-1}$ | 10.6 | 5.2 |
| X-rays | 2.0 Gy | 2.4 | <0.5 |

In order to rule out the possibility that the induction of CMD resistance produced a concomitant decrease in the antigenicity in the HX78Cy line, a series of immunization studies were performed. Tumours were excised and chopped finely into a tumour brei. This was sterilized using a dose of 100 Gy $^{60}Co$ irradiation and volumes of 0.2 ml were injected i.m. into the hind limb of mice; two immunizations were given, separated by 7 days. Figure 4 shows the tumour growth data for the parent (HX78) line and the CMD resistant (HX78Cy) line, each given either no immunization or following immunization with either the resistant or sensitive tumour line material. In neither case was there evidence that this type of immunization inhibited tumour growth.

### Discussion

The present experiments demonstrate the two main forms of drug resistance, inherent and induced, in human small cell bronchial carcinoma.

The line designated HX72 came from a patient after relapse following chemotherapy. The poor partial response to combination chemotherapy in the patient coupled with the evidence that the xenografts were also relatively drug resistant (Shorthouse *et al.*, 1980, and the data presented here) indicate that in this case inherent resistance to CMD and MeCCNU was stable on xenografting and did not disappear through 20 xenograft passages. HX72 was also relatively resistant to melphalan yet was found to have a similar uptake of radioactively labelled melphalan when compared with a more sensitive xenograft (HX88). These results are in keeping with other reports on melphalan uptake in human tumours of different chemosensitivity (Wist *et al.*, 1981; Parsons *et al.*, 1981).

Induced resistance to chemotherapy is a variable phenomenon. The two small cell bronchial carcinoma lines, both taken from previously untreated patients, showed a similar responsiveness as xenografts when treated with identical doses of CMD. However, under the same schedule of repeated drug treatments, resistance progressively developed in one line (HX78) but not in the other (HX88). Concurrent experiments with doses of MeCCNU that were judged to give a similar level of response, in each of the 3 xenografts, did not lead to the induction of resistance. In the case of HX78 where resistance was induced against CMD but not against MeCCNU, the initial treatment (of the repeat treatment schedule) produced a larger response with CMD than with MeCCNU. Comparison of the relative ability of these two drugs to induce resistance is therefore difficult.

The 8-fold reduction in response to CMD of HX78 took over a year to achieve and even after this protracted period of treatment the response was not completely abolished with the drug dose employed. This finding may possibly be explained by properties specific to the model system, chemotherapy schedule or the tumour's inherent inability to rapidly alter its level of response. However, use of other schedules, drugs and tumours suggests that resistance is not readily induced in small-cell lung cancer xenografts. Other laboratory investigations of human tumours have shown that resistance may take many months to induce (Parsons & Morrison, 1978; Ohnoshi *et al.*, 1982) though others have taken very much shorter times (Beck *et al.*, 1979; Houghton *et al.*, 1981). Hodgkins Disease patients relapsing after achieving an initial CR with MOPP, when treated again with MOPP achieved a second CR in 56% of cases studies by De Vita's Group (Fisher *et al.* 1977).

The evidence for stability in the inherently resistant HX72 contrasts with the data on HX78 (Figure 2) which show that the resistance induced in this xenograft line largely disappeared within two passages without treatment. It therefore appears that drug resistance in HX72 and HX78Cy was basically different in type. The nature of this difference remains to be elucidated. The lack of stability in HX78Cy might imply that some sort of adaptive change had occurred. It is also possible that HX78 contained both resistant and sensitive elements from the onset and that the sensitive component was not completely eradicated by treatment. Some form of growth advantage for the sensitive cells may have allowed them to grow out when treatment was discontinued.

Non-stable resistance to methotrexate in small cell bronchial carcinoma cells has also been reported recently (Curt *et al.*, 1983). There was evidence that in this case the explanation lay in the location of genes coding for dihydrofolate reductase on double minute chromosomes which, since they lack centromeres, could be progressively lost by failure to segregate during cell division. Unstable resistance in human fibrosarcoma and epidermal carcinoma cells to interferon has also been reported (Lin *et al.*, 1982). These results contrast with a large body of data that document the stability of drug-induced resistance (Ohnoshi *et al.*, 1982; Houghton *et al.*, 1981; Parsons *et al.*, 1982).

The clinical implications of these observations are that the occurrence of induced drug resistance may vary from one patient to another and perhaps from one treatment to another. It may take a long time to induce and possibly be lost quickly. It therefore may not always be right to abandon a treatment that initially was effective. This could be an area where the availability of a rapid *in vitro* chemosensitivity test would offer significant clinical advantages.

The data shown in Table I indicate that the resistant line was also cross resistant to each of the other agents tested, including radiation. This is therefore a further reason why switching from the first choice drug treatment would in this tumour probably be unwise.

Immunohistochemical studies were performed using an antikeratin antibody which demonstrated the presence of keratin in HX72 and HX78Cy, the inherent and the induced resistant lines, but essentially none in the two sensitive lines (HX78 and HX88). The results of these investigations and further tissue analysis will be presented in more detail in a separate communication. These results, taken with the cross-resistance data, lead to a possible mechanism for the drug resistance in these small-cell bronchial cancer xenografts. Although conventional light microscopy examination of the original HX72 and HX78 tumours was reported as pure small-cell carcinoma, it seems likely that they contained squamous carcinoma elements or the potential for their production. In the case of HX78 these non-small-cell components were insufficiently developed to influence the initial response to CMD but could be induced by repeated drug treatment.

### References

BECK, W.T., MUELLER, T.J. & TANZER, L.R. (1979). Altered surface membrane glycoproteins in Vinca Alkaloid-resistant human leukaemic lymphoblasts. *Cancer Res.*, **39**, 2070.

CURT, G.A., CARNEY, D.N., COWAN, K.H. & 6 others (1983). Unstable methotrexate resistance in human small-cell carcinoma associated with double minute chromosomes. *N. Engl. J. Med.*, **308**, 199.

FISHER, R.I., DeVITA, V.T., HUBBARD, S.M. & YOUNG, R.C. (1977). Prolonged disease free survival in Hodgkin's Disease following reinduction with MOPP. *Proc. ASCO*, **18**, 318.

HOUGHTON, J.A., HOUGHTON, P.J., BRODER, G.M. & GREEN, A.A. (1981). Development of resistance to vincristine in a childhood rhabdomyosarcoma growing in immune-deprived mice. *Int. J. Cancer*, **28**, 409.

KOPPER, L. & STEEL, G.G. (1975). The therapeutic response of three human tumour lines maintained in immune-suppressed mice. *Cancer Res.*, **35**, 2704.

LIN, S.L., GREENE, J.J., TS'O, P.O.P. & CARTER, W.A. (1982). Sensitivity and resistance of human tumour cells to interferon and rIn·rCn. *Nature*, **287**, 417.

OHNOSHI, T., OHNUMA, T., TAKAHASHI, I., SCANLON, K., KAMEN, B.A. & HOLLAND, J.F. (1982). Establishment of methotrexate-resistant human acute lymphoblastic leukaemia cells in culture and effects of folate antagonists. *Cancer Res.*, **42**, 1655.

PARSONS, P.G., CARTER, F.B., MORRISON, L. & Sr REGIUS MARY (1981). Mechanism of melphalan resistance developed *in vitro* in human melanoma. *Cancer Res.*, **41**, 1525.

PARSONS, P.G. & MORRISON, L. (1978). Melphalan-induced chromosome damage in sensitive and resistant human melanoma cell lines. *Int. J. Cancer*, **21**, 438.

PARSONS, P.G., SMELLIE, S.G., MORRISON, L.E. & HAYWARD, I.P. (1982). Properties of human melanoma cells resistant to 5-(3',3'-Dimethyl-1-triazeno) imidazole-4-carboxamide and other methylating agents. *Cancer Res.*, **42**, 1454.

SHORTHOUSE, A.J., PECKHAM, M.J., SMYTH, J.F. & STEEL, G.G. (1980). The therapeutic response of bronchial carcinoma xenografts: a direct patient-xenograft comparison. *Br. J. Cancer*, **41** (Suppl. IV) 142.

STEEL, G.G., COURTENAY, V.D. & ROSTOM, A.Y. (1978). Improved immune-suppression techniques for the xenografting of human tumours. *Br. J. Cancer*, **37**, 224.

STEUART, C.D. & BURKE, P.J. (1971). Cytidine deaminase and the development of resistance to arabinosyl cytosine. *Nature* (*New Biol.*) **233**, 109.

VALERIOTE, F.A., BRUCE, W.R. & MEEKER, B.E. (1968). Synergistic action of cyclophosphamide and 1,3-Bis(2-chloroethyl)-1-nitrosourea on a transplanted murine lymphoma. *J. Natl Cancer Inst.*, **40**, 935.

WIST, E., MILLAR, J. L. & SHORTHOUSE, A.J. (1981). Melphalan uptake in relation to vascular and extracellular space of human lung tumour xenografts. *Br. J. Cancer*, **43**, 458.